# Supplementary material for: Reconciling Mining with the Conservation of Cave Biodiversity: A Quantitative Baseline to Help Establish Conservation Priorities
Source: PLoS One. 2016 Dec 20;11(12):e0168348. doi: 10.1371/journal.pone.0168348 (PMC5173368; doi:10.1371/journal.pone.0168348)
Supplement: S1 Dataset — (ZIP) [file pone.0168348.s002.zip › Taxa/Serra Norte/SN_2013/N4E/Lista N4E-49.pdf]

CAVIDADE N4E-0049

Classe	Ordem	Fam/Outros	Gên/Outros	Espécie	S-U	U-S	Zon.
Annelida	Oligochaeta			jovem		1	E
Arachnida	Araneae	Corinnidae		jovem		1	E
Arachnida	Araneae	Drymusidae		jovem		X	E
Arachnida	Araneae	Filistatidae		sp.1	X	X	E
Arachnida	Araneae	Pholcidae	*Leptopholcus*	sp.1		X	E
Arachnida	Araneae	Pholcidae	*Mesabolivar*	sp.1		X	E
Arachnida	Araneae	Pholcidae		jovem	X	X	E
Arachnida	Araneae	Scytodidae	*Scytodes*	jovem		1	E
Arachnida	Pseudoscorpiones	Olpiidae		sp.1		X	E
Chilopoda	Scolopendromorpha	Scolopocryptopidae	*Tidops*	jovem		1	E
Diplopoda	Polydesmida			jovem		X	E
Entognatha	Diplura	Campodeidae		sp.1		X	E
Insecta	Blattodea	Polyphagidae		jovem		1	E
Insecta	Coleoptera			jovem		X	E
Insecta	Diptera	Dolichopodidae		sp.	X		E
Insecta	Diptera	Drosophilidae		sp.	X		E
Insecta	Diptera	Psychodidae	Phlebotominae	sp.		X	E
Insecta	Diptera	Tipulidae	Tipulinae	sp.	X	X	E
Insecta	Heteroptera	Lygaeidae		jovem		X	E
Insecta	Heteroptera	Reduviidae	Reduviinae	sp.	1	2	E
Insecta	Homoptera	Cixiidae		sp.3	X		E
Insecta	Hymenoptera	Formicidae	*Acromyrmex*	sp.1	X		E
Insecta	Hymenoptera	Formicidae	*Camponotus*	sp.1		X	E
Insecta	Hymenoptera	Formicidae	*Camponotus*	sp.2	X		E
Insecta	Hymenoptera	Formicidae	*Crematogaster*	sp.1	X		E
Insecta	Hymenoptera	Formicidae	*Pachycondyla*	sp.2		X	E
Insecta	Hymenoptera	Formicidae	*Pheidole*	sp.1	X		E
Insecta	Hymenoptera	Formicidae	*Trachymyrmex*	sp.1	X		E
Insecta	Isoptera	Rhinotermitidae	*Heterotermes*	sp.		X	E
Insecta	Isoptera	Termitidae	*Nasutitermes*	sp.	X	X	E
Insecta	Lepidoptera	Noctuoidea		sp.5		18	E
Insecta	Neuroptera	Myrmeleontidae		jovem	1	2	E
Insecta	Orthoptera	Phalangopsidae	*Phalangopsis*	sp.	3		E
Insecta	Psocoptera	Ptiloneuridae	*Triplocania*	sp.9	X	X	E
Insecta	Siphonaptera	Leptopsyllidae		sp.1		X	E
Malacostraca	Isopoda	Dubioniscidae		sp.1		X	E
Amphibia	Anura	Strabomantidae	*Pristimantis*	cf. *fenestratus*	1		E
